# Serotyping, MLST, and Core Genome MLST Analysis of *Salmonella enterica* From Different Sources in China During 2004–2019

**DOI:** 10.3389/fmicb.2021.688614

**Published:** 2021-09-16

**Authors:** Shigan Yan, Wencheng Zhang, Chengyu Li, Xu Liu, Liping Zhu, Leilei Chen, Baowei Yang

**Affiliations:** ^1^Shandong Provincial Key Laboratory of Bioengineering, School of Bioengineering, Qilu University of Technology, Shandong Academy of Sciences, Jinan, China; ^2^Institute of Agro-Food Sciences and Technology, Shandong Academy of Agricultural Sciences, Jinan, China; ^3^College of Food Science and Engineering, Northwest A&F University, Yangling, China

**Keywords:** *Salmonella enterica*, whole genome sequencing, serotype, MLST, cgMLST

## Abstract

Salmonella enterica (*S. enterica*) is an important foodborne pathogen, causing food poisoning and human infection, and critically threatening food safety and public health. *Salmonella* typing is essential for bacterial identification, tracing, epidemiological investigation, and monitoring. Serotyping and multilocus sequence typing (MLST) analysis are standard bacterial typing methods despite the low resolution. Core genome MLST (cgMLST) is a high-resolution molecular typing method based on whole genomic sequencing for accurate bacterial tracing. We investigated 250 *S. enterica* isolates from poultry, livestock, food, and human sources in nine provinces of China from 2004 to 2019 using serotyping, MLST, and cgMLST analysis. All *S. enterica* isolates were divided into 36 serovars using slide agglutination. The major serovars in order were Enteritidis (31 isolates), Typhimurium (29 isolates), Mbandaka (23 isolates), and Indiana (22 isolates). All strains were assigned into 43 sequence types (STs) by MLST. Among them, ST11 (31 isolates) was the primary ST. Besides this, a novel ST, ST8016, was identified, and it was different from ST40 by position 317 C → T in *dnaN*. Furthermore, these 250 isolates were grouped into 185 cgMLST sequence types (cgSTs) by cgMLST. The major cgST was cgST235530 (11 isolates), and only three cgSTs contained isolates from human and other sources, indicating a possibility of cross-species infection. Phylogenetic analysis indicated that most of the same serovar strains were putatively homologous except Saintpaul and Derby due to their multilineage characteristics. In addition, serovar I 4,[5],12:i:- and Typhimurium isolates have similar genomic relatedness on the phylogenetic tree. In conclusion, we sorted out the phenotyping and genotyping diversity of *S. enterica* isolates in China during 2004–2019 and clarified the temporal and spatial distribution characteristics of *Salmonella* from different hosts in China in the recent 16 years. These results greatly supplement *Salmonella* strain resources, genetic information, and traceability typing data; facilitate the typing, traceability, identification, and genetic evolution analysis of *Salmonella*; and therefore, improve the level of analysis, monitoring, and controlling of foodborne microorganisms in China.

## Introduction

*Salmonella enterica* (*S. enterica*) is one of the primary foodborne pathogens to cause food poisoning and human infection (Zhang et al., 2015). Foodborne salmonellosis is an important public health concern worldwide, and it annually causes about 115 million infections and 370,000 deaths globally (Seif et al., 2018). The primary sources of *S. enterica* foodborne infection include poultry eggs, meats, and their derived products (Arthur et al., 2008). Therefore, it is crucial to monitor *Salmonella* from animal food, especially poultry eggs, and their derived food products (Mezal et al., 2014).

Accurate typing and tracing are essential for microbial epidemiological investigation, food safety, and public health. Bacterial typing methods include phenotyping and genotyping. Among them, serotyping and multilocus sequence typing (MLST) are the most frequently used.

Serotyping identification has become the general standard method for *Salmonella* traceability and phenotypic classification for nearly 100 years. Serotype classification is based on serum agglutination tests of bacterial O and H antigens using the White–Kauffmann–Le Minor (WKL) scheme, which is adopted worldwide by public health organizations (Mezal et al., 2014). Up to now, more than 2610 serovars (also serotypes) of *Salmonella* have been documented globally (Monte et al., 2021). Moreover, *Salmonella* serotypes usually relate to their host adaptation and virulence, and the change in serotype proportion could reflect the epidemic status, so serotyping plays an essential role in *Salmonella* surveillance and outbreak investigations (Fierer and Guiney, 2001; Kumar et al., 2009; Dera-tomaszewska, 2012; Barbour et al., 2015). In the past, more attention has been paid to serovars Typhimurium and Enteritidis (Almeida et al., 2018).

In recent years, gene sequencing-based typing assays have been rapidly developed for bacterial tracing with vigorous vitality, including pulsed-field gel electrophoresis, MLST, core genome MLST (cgMLST), whole genome multilocus sequence typing (wgMLST), and whole gene single nucleotide polymorphism (wgSNP) (Liu et al., 2016, 2019; Radomski et al., 2019; Tiba-Casas et al., 2019). Among these molecular typing methods, MLST was developed to establish analytical microorganism typing (Maiden et al., 1998), recognize evolutionary relationships of *Salmonella* (Achtman et al., 2012; Ashton et al., 2016; Mairi et al., 2020; Zhang et al., 2020), and determine clonal isolate distributions across various environments and hosts (Wang M. et al., 2020; Zhao et al., 2020). In addition, there is a strong correlation between serotypes and sequence types (STs) of *Salmonella* (Wang X. et al., 2020). However, MLST cannot obtain accurate traceability of Typhimurium and I 4,[5],12:i:- because *Salmonella* isolates of these two serovars are often divided into the same STs (Possebon et al., 2020).

With the widespread extension of whole genomic sequencing (WGS), WGS-based high-resolution molecular subtyping methods have become popularized in outbreak investigation and bacterial tracing. As a kind of WGS-based subtyping method, cgMLST has high accuracy and can divide strains with minor sequence differences into different cgMLST sequence types (cgSTs), providing a powerful typing approach for molecular epidemiologic investigations. It is most commonly applied for foodborne disease surveillance in the public health area (Yoshida et al., 2016; Vincent et al., 2018; Ben Hassena et al., 2021). cgMLST is proven to be an adequate tool for cluster definition and has become a routine means in many countries and laboratories (Mellmann et al., 2011; Sabat et al., 2017; Simon et al., 2018; Robertson et al., 2019; Wang Y. J. et al., 2020; Hyeon et al., 2021).

In this article, we examine the serotypes, MLST, and cgMLST of *S. enterica* isolates from different sources in nine provinces in China from 2004 to 2019 to investigate their phenotyping and genotyping diversities and genetic relationships.

## Materials and Methods

### *Salmonella enterica* Isolates

**FIGURE 1:**
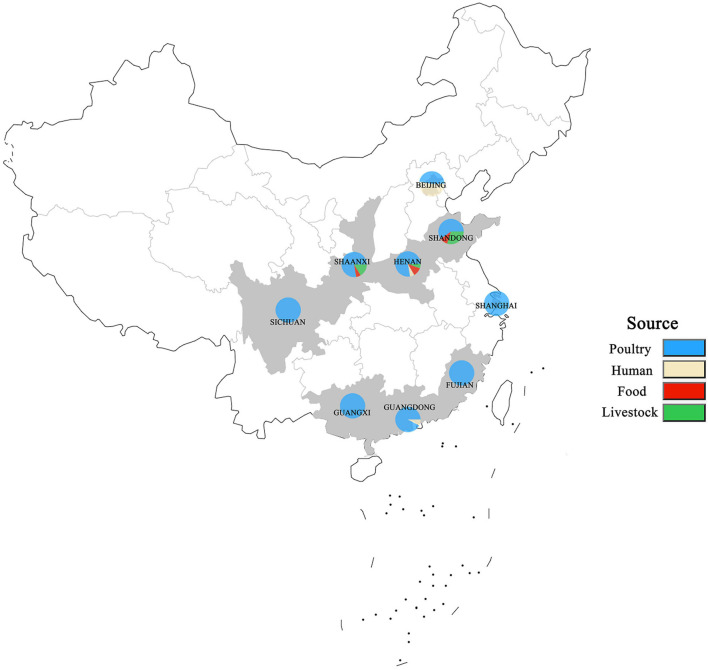
Distribution of *S. enterica* isolates in China from 2004 to 2019. The locations where the strains were isolated are shown on the map. The isolates obtained from humans, poultry, livestock, and food are labeled in different colors.

A total of 250 *S. enterica* isolates tested were from different sources in nine provinces in China from 2004 to 2019 except for 2005 and 2013 (Figure 1). The nine provinces, including Guangdong, Guangxi, Fujian, Sichuan, Shaanxi, Henan, Shandong, Shanghai, and Beijing, were major animal-breeding regions in China. The numbers of *S. enterica* isolates from different sources, years, or provinces were not equivalent. The details of these *S. enterica* isolates are shown in Supplementary Table 1. Out of these 250 isolates, 197 were from poultry-derived products, 31 from human, 15 from livestock meat, and 7 from infant nutrition rice formula (food); 16 were isolated in 2004, 9 in 2006, 4 in 2007, 11 in 2008, 31 in 2009, 105 in 2010, 27 in 2011, 13 in 2012, 1 in 2014, 10 in 2015, 5 in 2016, 5 in 2017, 7 in 2018, and 5 in 2019; 13 were separated in Guangdong, 19 in Guangxi, 11 in Fujian, 28 in Sichuan, 67 in Shaanxi, 18 in Henan, 10 in Shandong, 29 in Shanghai, and 55 in Beijing.

Among the 250 *S. enterica* isolates, 219 strains were isolated from animal products or nutrition rice formula samples. All food samples were immediately homogenized and subjected to *Salmonella* isolation following the standard protocol as previously described (World Health Organization (WHO), 2003). In short, the sample homogenates were added into selenite cysteine broth and incubated at 35°C for 24 h to selectively enrich *Salmonella*. Each enriched broth was streaked onto *Salmonella Shigella* and xylose lysine deoxycholate agar plates and incubated at 37°C for 24 h. Presumptive *Salmonella* colonies were picked and cultured in triple sugar iron agar media and then systematically identified by microbiological, biochemistry, and 16S rDNA sequencing analysis to confirm *Salmonella* strains. Thirty-one *Salmonella* isolates from humans were isolated and identified by the Beijing Center for Disease Prevention and Control. All identified *Salmonella* isolates were stored in 25% (v/v) glycerol at −80°C in our lab and reproduced periodically.

### Serotyping by Slide Agglutination and Prediction by Genome

*Salmonella enterica* isolates were cultured in nutrient broth at 37°C overnight. A drop of fermentation broth was taken on glass slides to test somatic O antigen by slide agglutination. Meanwhile, each strain was grown on Swarm agar plates at 37°C overnight, and single colonies were picked to test phases 1 and 2 of H antigens by slide agglutination. Diagnostic sera for *Salmonella* antigens were purchased from Tianrun Bio-Pharmaceutical Co. (Ningbo, China) and SandA Reagents Lab Ltd. (Bangkok, Thailand). *Salmonella* serotyping was classified by the WKL scheme. Additionally, O antigen, H antigen, and serovars were predicted based on *Salmonella* genomes using *Salmonella In Silico* Typing Resource (SISTR^1^).

### Bacterial Genome Sequencing and Genomic Assembly

The genomic DNA of each *S. enterica* isolate was extracted with the sodium dodecyl sulfate method using the TIANamp Bacteria DNA Kit DP302-02 (TIANGEN, China) following the manufacturer’s instructions. The extracted genomic DNA quality and integrity were evaluated on 0.5–1% agarose gels, concentration was measured using a fluorimeter (MD2000H, Biofuture), and purity was determined with a spectrophotometer based on the ratio of OD_260_ to OD_280_ (OD_260_/OD_280_ ≥ 1.8). The sequence libraries were constructed using Illumina’s Nebnext Ultra DNA Library Prep Kit (NEB, United States). According to different attribute sequences, each sample was assigned with an index code. In brief, the DNA sample was first broken into approximately 350 bp fragments by sonication. After end repair, DNA fragments were ligated head-to-tail to a full-length adaptor for further PCR amplification. The PCR products were purified by the AMPure XP system, the size distribution of the libraries was analyzed by the Agilent bioanalyzer, and quantitative analysis was performed by real-time PCR.

The genomic DNA of 250 *S. enterica* isolates was sequenced using the Illumina NovaSeq PE150 platform at Beijing Novogene Bioinformatics Technology Co., Ltd. Considering the influence of low-quality data in the obtained raw sequencing data on the accuracy and reliability of subsequent information analysis, the original data were filtered to obtain the clean data. The specific processing steps included (1) removal of the reads containing low-quality bases (mass value ≤20) over a certain percentage (the default was 40%), (2) removal of reads containing a higher proportion of N (the default was 10%), (3) removal of sequences overlapping with adapters exceeding a certain threshold (the default was 15 bp) and with fewer than three mismatches, and (4) removal of data that might originate from the host after BLASTing against the host database.

The specific processing steps for genome assembly with clean data included (1) assembling with SOAP *de novo* software (Li et al., 2008) with different *K*-mers (the default was 107) first and then with the optimal *K*-mer after adjusting other parameters (-d -u -R -F, etc.) according to the project type, and the least scaffolds were chosen as the preliminary assembly result; (2) assembling with SPAdes software (Bankevich et al., 2012) with different *K*-mers (the default was 127) and then with the optimal *K*-mer according to the project type, and the assembly results as the least scaffolds were obtained; (3) assembling with Abyss software (Simpson et al., 2009) with the 64 nt *K*-mer to obtain the assembly results; (4) using the CISA software to integrate the above three assembly results, and only the assembly results with the least scaffolds were selected; (5) using GapCloser software to fill the gap of preliminary assembly results and remove the same lane pollution by filtering the reads with low sequencing depth (less than 0.35 of the average depth) to obtain the final assembly results; and (6) counting the final assembly result (without fragments below 500 bp) for gene prediction by GeneMarkS software (Besemer et al., 2001).

### MLST, cgMLST, and Phylogenetic Analysis

*Salmonella enterica* isolates were analyzed based on genomic sequences (clean data) using *in silico* MLST and cgMLST analysis on the EnteroBase online platform^2^ for *Salmonella*. Seven housekeeping gene loci, including *aroC*, *dnaN*, *hemD*, *hisD*, *purE*, *sucA*, and *thrA*, were chosen for MLST analysis of *Salmonella*. The neighbor-joining tree of *S. enterica* was established based on MLST information.

A *Salmonella* cgMLST v2 scheme comprising 3002 target loci of *Salmonella* was employed to analyze cgMLST based on genomic sequences, and neighbor-joining was used to make the dendrograms based on cgMLST information. *Salmonella* GrapeTree was constructed based on the above cgMLST scheme by EnteroBase GreepTree using NINJA neighbor-joining algorithm.

The tanglegram algorithm was applied to compare the MLST and cgMLST phylogenetic trees by placing trees side by side and drawing a straight line (or connector) between corresponding taxa (Scornavacca et al., 2011; Huson and Scornavacca, 2012). Although the algorithm can effectively reduce the number of intersections between connectors, the change of nodes in the evolution of these two types of phylogenetic trees are in the same direction and can lead to multiple short-distance intersections between connectors. If the two types of trees were identical and no connectors crossed, the cluster of phylogenetic tips remains unchanged.

## Results

### Serotyping Classification of *S. enterica* in China During 2004–2019

**FIGURE 2:**
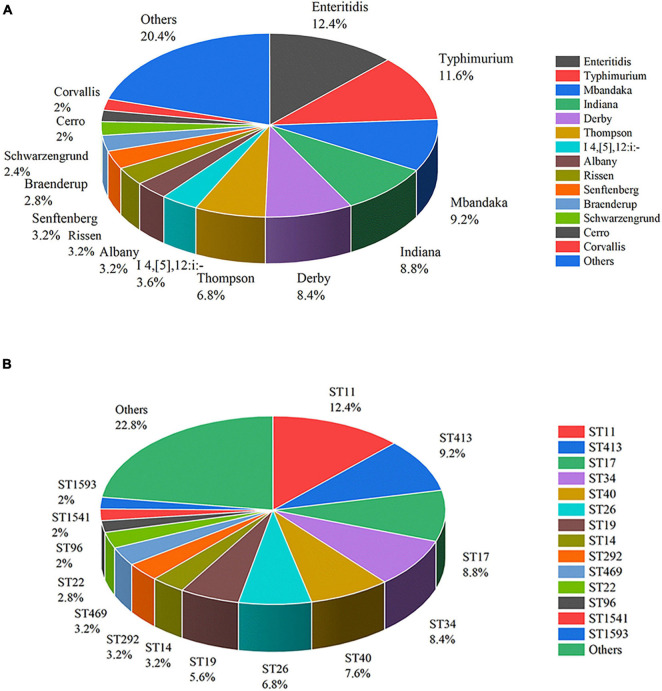
Distribution of *Salmonella enterica* isolates of serotypes **(A)** and STs **(B)**.

The 250 *S. enterica* strains tested in the study were divided into 36 serotypes by slide agglutination (Figure 2A). *Salmonella* serovar Enteritidis (*n* = 31) was the most common serotype in China from 2004 to 2019, followed by Typhimurium (*n* = 29), Mbandaka (*n* = 23), Indiana (*n* = 22), Derby (*n* = 21), Thompson (*n* = 17), Agona (*n* = 12), and I 4,[5],12:i:- (*n* = 9). Senftenberg, Rissen, and Albany accounted for 3.2% (*n* = 8), respectively. Braenderup and Schwarzengrund possessed seven and six isolates, respectively. Cerro and Corvallis both had five isolates. Blockley, Hadar, Infantis, Meleagridis, Newport, and Saintpaul each encompassed four isolates. Havana and Kentucky both had three isolates. Bovismorbificans, Hvittingfoss, Kottbus, and Stanley each contained two isolates. Anatum, Litchfield, London, Pomona, Potsdam, Tennessee, and Uganda each included one isolate. There were 19 serotypes observed in more than two provinces. Albany, Mbandaka, Enteritidis, I 4,[5],12:i:-, and Typhimurium appeared in three provinces.

The 250 *S. enterica* isolates were divided into different serotypes based on their sources (Supplementary Table 1). The 197 isolates from poultry belonged to 34 serotypes, among which Typhimurium had the most isolates (*n* = 24), followed in turn by Indiana (*n* = 20), Enteritidis (*n* = 20), Mbandaka (*n* = 18), Derby (*n* = 17), and Thompson (*n* = 16). The 31 isolates from humans belonged to 13 serotypes with Enteritidis accounting for the largest percentage with 32.26% (*n* = 10). The 15 isolates from livestock contained nine serotypes, among which Derby and Mbandaka each included four stereotypes. In addition, the seven isolates from foods belonged to seven different serotypes.

According to statistics of the isolation years, the number of isolates was different in each year with Typhimurium, Enteritidis, or I 4,[5],12:i:- being the most abundant ones during 2012–2019. Our investigations are similar to those recently reported; Enteritidis and Typhimurium are still major serovars of *Salmonella* from animal food or humans (Greig and Ravel, 2009; Hendriksen et al., 2011; Yang et al., 2019; Perry et al., 2020; Shen et al., 2020; Xin et al., 2021).

Comparative serotyping analysis of the 250 isolates showed that the serotypes of 245 (98%) isolates based on slide agglutination were consistent with the genome-based prediction, and only five isolates, QLULP2, QLUY902, QLUY914, QLUY931, and QLUY933, were different. Among them, serotype Typhimurium of QLULP2, QLUY902, QLUY914, and QLUY933 based on slide agglutination was wrongly predicted as I 4,[5],12:i:-, and the serotype I 4,[5],12:i:- of QLUY931 based on slide agglutination was wrongly predicted as Typhimurium. It is worth noting that Typhimurium and I 4,[5],12:i:- were rare in genome-based prediction by SISTR in our study. The results proved once again that *Salmonella* serotype prediction based on the genome is concordant with serovar by the serum agglutination test except Typhimurium and I 4,[5],12:i:-, so WGS-derived serotyping can replace the agglutination assay to some extent and be applied in the typing, traceability, and identification of *Salmonella*.

### *Salmonella* Draft Genome Analysis

The genomic reads of 250 *S. enterica* isolates were stored in the Sequence Read Archive (SRA) of the National Center for Biotechnology Information (NCBI). SRA serial numbers of the submitted 250 strains are shown in Supplementary Table 1.

A total of 9656 (≥500 bp) contigs varying from 9 to 92 with an average of 38.62 per genome were generated. The average draft genome size was 4.88 Mb, ranging from 4.35 to 5.48 MB. Likewise, the average G + C content observed was 52.04%. Functional annotation of all draft genomes predicted an average of 4692 genes, ranging from 4173 to 5334.

### *In silico* MLST Analysis of *S. enterica*

The 250 *Salmonella* isolates were divided into 43 STs and 37 eBurst groups (eBGs) using *in silico* MLST. However, ST3134 belonged to neither eBG. ST11 was the most common (*n* = 31), followed in turn by ST413 (*n* = 23), ST17 (*n* = 22), ST34 (*n* = 21), ST40 (*n* = 19), ST26 (*n* = 17), ST19 (*n* = 14), ST14 (*n* = 8), ST292 (*n* = 8), and ST469 (*n* = 8). ST distribution is shown in Figure 2B.

In addition, among the 43 STs identified, a novel ST named ST8016 (QLUY608) was obtained, and it differed from ST40 by 1 SNP in the *dnaN* locus at position 317 (C → T, named *dnaN*1076, compared to *dnaN*20). Compared with the ST40 *dnaN* locus, this mutation occurred in the second codon position, resulting in a non-synonymous change from C to Y. Our findings enrich MLST data of *Salmonella*, facilitating the typing, traceability, and identification of *Salmonella*.

Among the 43 STs, 25 STs were observed in more than two provinces and 18 STs in one province. ST17 and ST40 appeared in seven provinces. The results indicate that these two STs were prevalent and had the possibility of transregional infection.

Statistics of the isolation sources revealed that (1) the 31 isolates from humans consisted of 13 different STs, among which ST11 had the highest number of 10 isolates, followed by ST34 (*n* = 6); (2) the 197 isolates from poultry belonged to 37 STs, among which ST11 had the highest number (*n* = 22), followed in turn by ST413 (*n* = 21), ST17 (*n* = 16), ST40 (*n* = 16), and ST26 (*n* = 15); (3) the 15 isolates from livestock contained 9 STs, among which ST413 had the highest number (*n* = 4); and (4) the 7 isolates from food belonged to 7 different STs.

Statistics of isolation years found that the major ST of every year was different during 2004–2011. ST413 was the major ST in 2004, 2007, and 2011; ST11 was the major ST during 2012–2019 except 2014; and ST34 was the major ST from 2014 to 2019 except 2018. The results showed that the major ST of *Salmonella* was not constant in every year.

Comparison of MLST and serotyping showed that each ST only comprised one serotype except ST34. Nine isolates of I 4,[5],12:i:- and 12 isolates of Typhimurium belonged to ST34. In addition, four serovars included more than one ST: 29 isolates with serotype Typhimurium contained four STs (ST19, ST34, ST128, and ST1544), 21 isolates of Derby contained three STs (ST40, ST8016, and ST71), 4 isolates of Newport contained 2 STs (ST50 and ST3134), and 6 isolates of Schwarzengrund contained two STs (ST96 and ST241). The results represent that the accuracy of MLST was higher than that of serotyping.

### cgMLST Analysis of *Salmonella* Based on Genomic Sequences

A total of 3002 target genes were identified from the *S. enterica* genome using the EnteroBase cgMLST module. The cgMLST analysis of *S. enterica* isolates is shown in Supplementary Table 1. The cgMLST analysis revealed that the 250 *Salmonella* isolates were grouped into 185 cgSTs, all of which contained only one serotype. Among them, 243 isolates belonged to 182 novel cgSTs, and 7 belonged to three known cgSTs. Among the 185 cgSTs, cgST235530 contained the most abundant isolates, followed by cgST217495 (*n* = 6) and cgST234930 (*n* = 6). We comprehensively analyzed the WGS-based genotypes of *Salmonella* in China in the past 16 years and found many novel cgSTs, which enriched the genotype data resources and promoted the development of traceability level of *Salmonella*.

The relationship of cgST with the years, regions, and sources of isolation is shown in Table 1. Among the 185 cgSTs, 33 cgSTs contained more than two isolates, and 15 cgSTs contained strains from different years, provinces, or sources of isolation. In detail, 12 cgSTs isolates were from multiple provinces, 12 cgSTs from different years, and 5 cgSTs from different sources, representing the possibility of cross-outbreaking in different locations, hosts, or years. cgST236211, cgST236321, and cgST236324 contained multiple isolates from human and other sources, indicating a possibility of cross-species infection. In addition, 152 strains had unique cgSTs in this study.

**TABLE 1:** *Salmonella enterica* cgSTs from different years, provinces, or sources of isolation.

**cgST**	**Numbers**	**Source**	**Province**	**Years**
cgST42868	5	Poultry	Shaanxi, Fujian	2010, 2011
cgST217495	6	Poultry	Beijing, Shaanxi	2010, 2012
cgST234925	3	Poultry	Sichuan, Fujian	2010
cgST234927	3	Poultry	Sichuan, Shaanxi	2006, 2010
cgST234930	6	Poultry, food	Sichuan, Shaanxi	2006, 2007, 2010, 2012
cgST234932	3	Poultry	Sichuan, Fujian	2010
cgST235529	2	Poultry, food	Sichuan, Shaanxi	2006, 2010
cgST235530	11	Poultry	Shaanxi, Guangdong	2010, 2011
cgST235678	2	Poultry	Sichuan, Shaanxi	2010, 2011
cgST235686	2	Poultry	Guangxi, Shaanxi	2010, 2011
cgST235712	2	Poultry	Shaanxi, Shanghai	2006
cgST235797	2	Poultry	Guangdong, Shaanxi	2007, 2011
cgST236211	2	Poultry, human	Guangdong	2010
cgST236321	2	Poultry, human	Beijing	2008, 2012
cgST236324	2	Poultry, human	Beijing	2009, 2012

**FIGURE 3:**
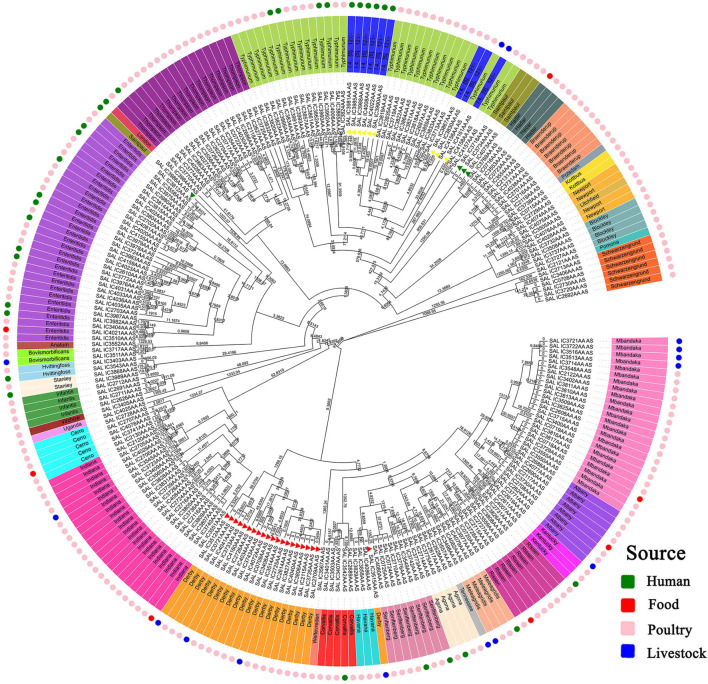
Core genome MLST-based phylogenetic tree of *S. enterica* isolates. The neighbor-joining algorithm phylogenetic tree was created by EnteroBase based on cgMLST information and visualized using Evolview. The numbers stand for Branchlength values. The isolates’ serotypes are labeled in color, and most serovars forms serovar-specific clades except Saintpaul, Derby, and I 4,[5],12:i:-, which are signaled by the same color triangles. I 4,[5],12:i:- and Typhimurium may have a close genetic and evolutionary relationship. Further determination of the evolutionary relationship between the abovementioned serovars will be furtherly carried out. Different colors are used in the outermost ring to indicate the separation source to distinguish the specificities of different isolates.

**FIGURE 4:**
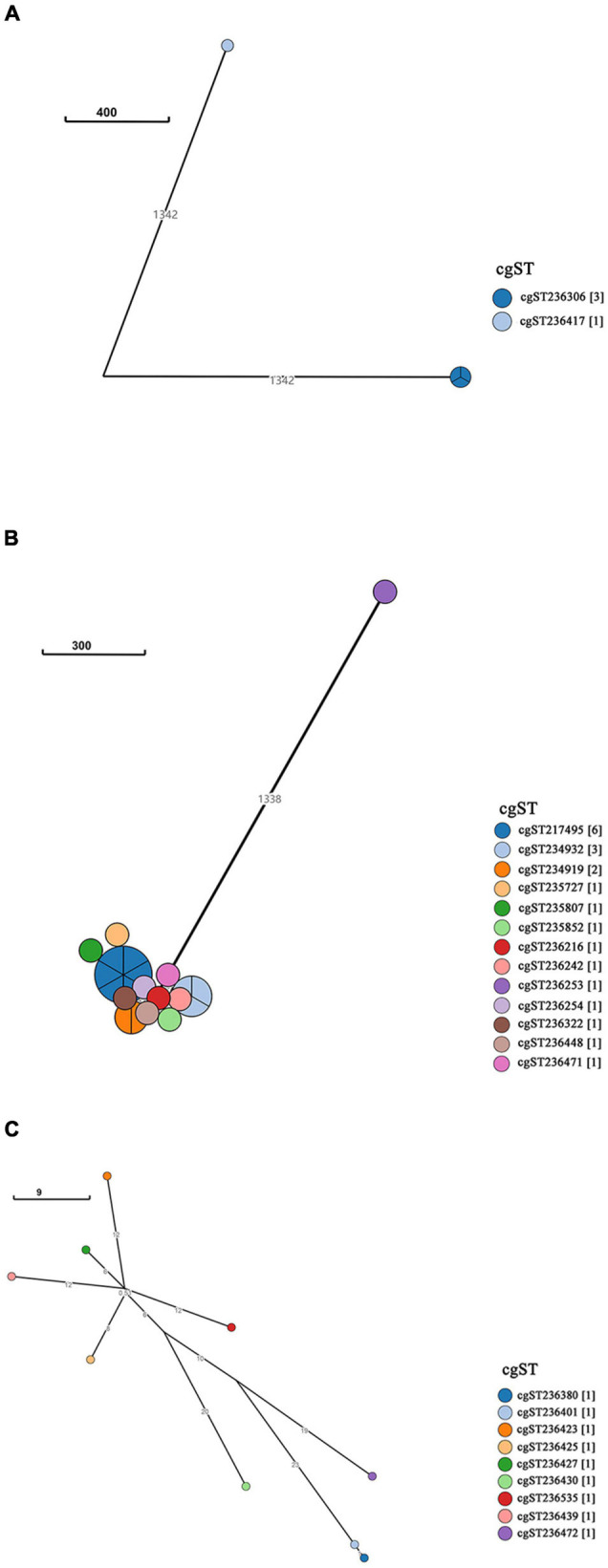
GrapeTree of *Salmonella* serovar Saintpaul, Derby, and I 4,[5],12:i:- isolates. GrapeTree constructed based on the 3002 core genes by EnteroBase GrapeTree using NINJA neighbor-joining algorithm. **(A)** GrapeTree among the four serovar Saintpaul isolates, where every node means different cgST and the line between two nodes indicates a long distance (≥1000); **(B)** GrapeTree of the 21 Derby and 1 cgST (cgST236253) isolates with a long genetic distance (≥1000) to other cgSTs; **(C)** GrapeTree of the nine serovar I 4,[5],12:i:- and nine cgST isolates with nodes indicating they all have a relatively close genetic distance (≤100).

A neighbor-joining tree was generated based on the 250 strains’ cgMLST information (Figure 3). Many serovars formed serovar-specific clades in this tree, suggesting that the same serovars were putatively homologous except that isolates of Saintpaul, Derby, and I 4,[5],12:i:- were heterologous. To further investigate the relationship of Saintpaul, Derby, and I 4,[5],12:i:- isolates, the GrapeTrees of each serovar were constructed based on cgMLST information (Figure 4). In the GrapeTrees, Saintpaul contained four isolates belonging to two clades with a long genetic distance, representing that Saintpaul was putatively polyphyletic (Figure 4A). Similarly, Derby contained 21 isolates that did not wholly cluster together. QLUY614 (cgST236253) had a long genetic distance to the other 20 isolates, suggesting Derby was putatively polyphyletic, too (Figure 4B). Differently, the nine isolates of serovar I 4,[5],12:i:- had close genetic distances in the GrapeTree even though they did not completely cluster together (Figure 4C). In a word, isolates of Saintpaul and Derby were putatively polyphyletic and characterized by multilineages (Yin et al., 2020).

**FIGURE 5:**
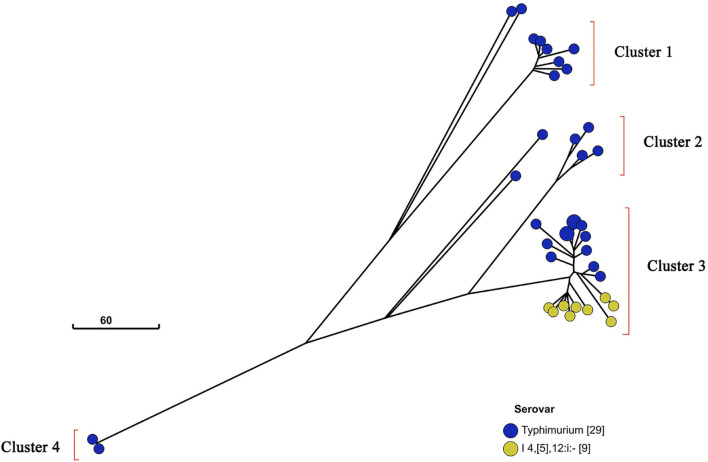
GrapeTree of *Salmonella* serovar I 4,[5],12:i:- and Typhimurium isolates. GrapeTree of nine serovar I 4,[5],12:i:- isolates and 29 serovar Typhimurium isolates constructed based on cgMLST information by EnteroBase GrapeTree function and neighbor-joining algorithm. These 38 isolates belong to four clusters in the tree, and all nine serovar I 4,[5],12:i:- isolates belong to cluster 3 with 12 serovar Typhimurium isolates, showing that *Salmonella* serovar I 4,[5],12:i:- and Typhimurium have a closer genetic relationship.

Additionally, I 4,[5],12:i:- and Typhimurium in a similar genetic distance may have a near genetic relationship (Figure 3). To further investigate the relatedness of I 4,[5],12:i:- and Typhimurium, their GrapeTree was constructed based on cgMLST information of serovar I 4,[5],12:i:- (*n* = 9) and Typhimurium (*n* = 28) (Figure 5). In the GrapeTree, Typhimurium isolates were clustered into eight highly clonal clades and four highly clonal clusters (clusters 1–4), and nine isolates of I 4,[5],12:i:- and 12 isolates of Typhimurium were clustered into cluster 4. The low homologous diversity within I 4,[5],12:i:- and Typhimurium in combination with the above cgST tree on coherent clades indicate that these two serovars might have similar genomic sequences.

In addition, only three isolates from patients were in the same genetic position with other strains in the evolutionary tree. As mentioned, isolates of cgST236211, cgST236321, and cgST236324 were in the same genetic position in the tree: QLULY5 (human, Beijing, 2009) with QLULR4 (poultry, Beijing, 2012) of Thompson; QLULY1 (human, Beijing, 2008) with QLULR2 (poultry, Beijing, 2012) of Senftenberg; and QLULN4 (human, Guangdong, 2010) with QLUY510 (poultry, Guangdong, 2010) of Hvittingfoss. There was a strong homology among these isolates and a possibility of cross-species infection in patients although significant differences existed in some isolates from different years of isolation.

Analysis of isolates’ clustering position in the evolutionary tree showed isolates of Hadar, I 4,[5],12:i:-, Saintpaul, Kentucky, and Mbandaka had a strong correlation between genetic position and source (Figure 3). Among the three Kentucky isolates, QLULA8 (Guangxi, 2010) and QLULA5 (Beijing, 2010) isolates from poultry were putatively homologous and had a long genetic distance to the isolate QLUF123 (Beijing, 2018) from patients. What is more, among the 13 Mbandaka isolates, four from livestock were in the same genetic position in the evolutionary tree with high homology and had a long genetic distance to other isolates of different sources. Obviously, serovar I 4,[5],12:i:- isolates from the same hosts were at an adjacent genetic position in the evolutionary tree.

### Major Serovars and cgSTs of *Salmonella* Isolates From Different Sources, Years, and Provinces

A total of 25 major cgSTs and 26 major serovars appeared among the 250 *Salmonella* isolates from different sources in nine provinces during 2004–2019. Among them, four major serovars from livestock in Fujian province in 2007 and 2011 were clustered in major cgSTs. In detail, 11 major cgSTs belonged to major serovars. For example, four isolates of cgST236240 in 2004 belonged to Mbandaka, and the major serovars in 2004 were Derby (*n* = 4) and Mbandaka (*n* = 4). Obviously, the major serovar Typhimurium clustered with 19 isolates in 2009, but the major cgST was cgST236385 (*n* = 3, Havana). Meanwhile, the major serovar from poultry was Typhimurium (*n* = 28), and the major cgST was cgST235530 (*n* = 11, Mbandaka). The 28 Typhimurium isolates were divided into 26 different cgSTs and seven isolates from food belonged to seven serovars and seven cgSTs. The major serotype was always Typhimurium, Enteritidis, or I 4,[5],12:i:- during 2015–2019. However, these three serovar isolates possessed different cgSTs. Major cgSTs and major serovars of *Salmonella* isolates from different sources in different years and provinces are shown in Table 2.

**TABLE 2:** Major cgSTs and major serovars of *S. enterica* isolates.

	**Number of strains**	**Major cgSTs (no., serovar)**	**Major serovars (no.)**
**Year**			
2004	16	cgST236240 (4, Mbandaka)	Derby (4), Mbandaka (4)
2006	9	cgST235712 (2, Infantis)	Infantis (2), Thompson (2)
2007	4	cgST234930 (2, Mbandaka)	Mbandaka (2)
2008	11	cgST236457 (2, Meleagridis)	Corvallis (3)
2009	32	cgST236385 (3, Havana)	Typhimurium (19)
2010	105	cgST42868 (4, Braenderup)	Indiana (12)
		cgST234918 (4, Schwarzengrund)	
		cgST234928 (4, Senftenberg)	
2011	27	cgST235530 (9, Mbandaka)	Mbandaka (9)
2012	13	cgST217495 (4, Derby)	Enteritidis (6)
2014	1	–	–
2015	10	–	Typhimurium (4)
2016	5	–	Enteritidis (2), I 4,[5],12:i:- (2)
2017	5	–	Enteritidis (2), I 4,[5],12:i:- (2)
2018	7	–	Enteritidis (2), Typhimurium (2)
2019	5	–	Enteritidis (2), I 4,[5],12:i:- (2)
**Province**			
Beijing	55	cgST217495 (3, Derby)	Enteritidis (15)
Fujian	11	cgST234918 (4, Schwarzengrund)	Schwarzengrund (4)
Guangdong	13	cgST236218 (2, Corvallis)	Mbandaka (3)
		cgST236211 (2, Hvittingfoss)	
		cgST235530 (2, Mbandaka)	
Guangxi	19	cgST235719 (2, Cerro)	Albany (3)
		cgST234919 (2, Derby)	Cerro (3)
		cgST236457 (2, Meleagridis)	Newport (3)
		cgST236458 (2, Newport)	
Henan	18	–	Enteritidis (3)
Shandong	10	cgST236436 (2, Enteritidis)	Enteritidis (5)
Shanghai	29	cgST236385 (3, Havana)	Typhimurium (19)
Shaanxi	67	cgST234930 (9, Mbandaka)	Mbandaka (18)
Sichuan	28	cgST234928 (4, Senftenberg)	Thompson (5)
**Source**			
Food	7	–	–
Human	31	–	Enteritidis (10)
Livestock	15	cgST236240 (4, Mbandaka)	Mbandaka (4)
Poultry	197	cgST235530 (11, Mbandaka)	Typhimurium (28)

*“–” means no major serovars or major cgSTs.*

### Comparative Analysis of MLST and cgMLST Within *S. enterica* Isolates

**FIGURE 6:**
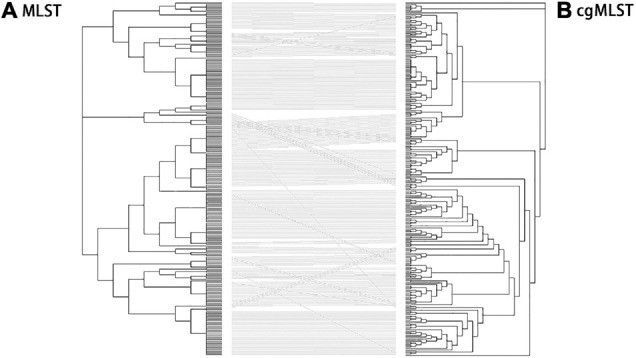
Comparison of phylogenies by MLST **(A)** and cgMLST **(B)** based on genomic sequences of 250 *Salmonella* isolates. Tanglegram linking tips with the same label to each other *via* a straight line produced within dendroscope 3 of 250 *Salmonella* isolates. By comparing of N-J trees by MLST and cgMLST, clustering in the two trees was mostly congruent although a few sections show several inversions. This led to some isolates being clustered at the edge of one tree, moving to the center of the other. In addition, the tree by cgMLST was more detailed than that by MLST.

Identification of all loci present within *Salmonella* isolates was performed using MLST and cgMLST schemes. The 250 *S. enterica* isolates in China from 2004 to 2019 were used to generate a tanglegram for a visual comparison of cgMLST and MLST (Figure 6). Most of the straight lines were parallel, and only some minor straight lines crossed with others, indicating that the majority of STs were parallel to cgSTs. This phenomenon may give rise by differences between the located deeper internal nodes within these two phylogenies. By comparison, most of the isolates were grouped into the same clusters by MLST and cgMLST.

Further analysis shows that the crosslines were mainly due to the differences in the genetic relationships between some different serovar isolates in the evolutionary tree established by MLST and cgMLST. By comparison, the following serovar isolates had close genetic relationships in the MLST-based neighbor-joining tree but not in the cgMLST-based neighbor-joining tree: (1) 1 isolate of Virchow (QLUY603) with 5 Corvallis isolates, (2) 1 isolate of Weltevreden (QLULO6) with all 23 Mbandaka isolates and 8 Albany isolates, (3) 3 isolates of Kentucky with all 8 Rissen isolates, (4) f isolates of Cero with 1 Saintpaul isolate (QLUF115), and (5) 5 isolates of Saintpaul and all 31 Enteritidis isolates. Although MLST and cgMLST clustered two isolates of Stanley (QLUF119, QLUZ101) and two isolates of Hvittingfoss (QLULN4, QLUY510) together, the MLST-based neighbor-joining tree indicated one Virchow isolate (QLUY603) is closely related with five Corvallis isolates, and the cgMLST-based neighbor-joining tree placed them at a longer genetic distance. However, the majority of *Salmonella* isolates were grouped into the same clusters by both MLST and cgMLST. Obviously, the evolutionary tree by cgMLST was more detailed than that by MLST.

## Discussion

In recent years, with increasing demand for food safety, the detection of foodborne *Salmonella* becomes greatly important, and *Salmonella* from food is tested more intensively worldwide (Yang et al., 2019). *Salmonella* typing is essential for microbial determination, epidemiological investigation, and outbreak tracing. Serotyping and MLST are the gold standard bacterial typing methods. However, they both could not accurately trace because they are relatively low-resolution. Thus, it is significant to establish high-resolution and reliable subtyping methods for tracing epidemic strains in pathogenic outbreaks (Jolley et al., 2012).

Serotyping by slide agglutination has been widely adopted for *Salmonella* classification for nearly 100 years. In this study, we serotyped 250 *Salmonella* isolates from different sources in China in the past 16 years, and the results show all strains tested in the study were divided into 36 serotypes, and *Salmonella* serovars Enteritidis and Typhimurium were still the major serovars, which is similar to a recent report (Perry et al., 2020; Shen et al., 2020). Enteritidis is frequently isolated from animal food globally and is one of the most common serovars associated with human salmonellosis (Greig and Ravel, 2009; Hendriksen et al., 2011; Yang et al., 2019).

However, serotyping by slide agglutination has some shortcomings, such as being low-resolution, expensive, and time-consuming. In addition, this serological test requires well-trained personnel to operate it as well as high-quality sera; otherwise, it is prone to error in serotype determination because of artificial discrimination of differences in agglutination profiles and the incomplete phenomenon of H antigen expression (Zhou et al., 2020). However, it does not mean that serotyping will be rapidly replaced because it has become a traditional microbial phenotyping classification method by microorganists and public health organizations. Therefore, it will still play a fundamental role in bacterial tracing in the near future.

We also validated the serotyping by agglutination assay using SISTR prediction based on genomic sequences. The two methods are highly consistent with a consistency rate of 98%, which is similar to recent reports (Robertson et al., 2018; Uelze et al., 2019; Lyu et al., 2021). However, SISTR has a low identification for Typhimurium and I 4,[5],12:i:- in our research. This situation may be because SISTR only predicts serotypes based on related coding genes, and the results of slide agglutination tests are affected by many factors, such as gene mutation or inexpression.

Facing potential food safety risks, serological misidentification may endanger underestimating the occurrence of certain *Salmonella* serotypes outbreaks. The serotype prediction based on nucleic acid sequences plays an essential role in serotype validation. Therefore, it is essential to validate the slide agglutination assay. Several methods have been developed to predict serotypes based on nucleic acid sequences (Achtman et al., 2012; Inouye et al., 2014; Zhang et al., 2019). MLST is attractive because of being generated with easily replicated protocols and correlated well with the majority of lineages and serovars by means of eBGs. The advantages of MLST are more uniform, well relative with serotypes, and convenient to communicate by databases (Kimura, 2018).

The 250 *Salmonella* isolates were divided into 43 STs using *in silico* MLST with ST11 being the most abundant ST (*n* = 31). Among the 43 STs identified, a novel ST, ST8016 (QLUY608), differs from ST40 by 1 SNP in the *dnaN* locus at position 317 (C → T, named *dnaN*1076, compared with *dnaN*20). According to MLST typing, ST34 contains I 4, [5],12:i:- and Typhimurium. The method cannot distinguish I 4,[5],12:i:- from Typhimurium with the same STs because of similar genomic sequences. Furthermore, a GrapeTree was constructed using the genomic sequences of all isolates of I 4,[5],12:i:- (*n* = 9) and Typhimurium (*n* = 29). The results show that the isolates of the two serovars could not be completely clustered together: only nine isolates of I 4,[5],12:i:- and 12 isolates of Typhimurium belong to the same cluster and are in similar genetic positions on the evolutionary tree (Figure 5). However, the MLST approach may cause two main issues to predict serotype using SISTR: (1) a few STs do not have a serovar designation in the MLST database and (2) the unexpected identification of novel STs.

Molecular typing for *Salmonella* of the same serovars is essential in outbreak investigation and bacterial epidemiology. The increasing availability of analytical approaches for whole genome-based subtyping will continue to fuel the adoption of genomics in the context of epidemiological investigations. With the reduction of sequencing cost and the promotion of genome sequencing, a large amount of bacterial genome data has been generated, and it has become routine to trace microorganisms based on bacterial genomic sequences. Molecular typing methods based on WGS with high resolution and outstanding accuracy are used as routine bacterial trace analysis methods. Each has different advantages and drawbacks that determine its applicability and limitations. In recent years, cgMLST, wgMLST, and SNP have become innovative tracing tools (García-Soto et al., 2020; Gu et al., 2020; Monte et al., 2021). cgMLST well correlates with time and regions of bacterial isolates. Microorganism epidemiological investigation results found that the main pathogens may probably change during outbreaking, and these will be largely muted by conventional low-resolution typing methods (Quick et al., 2015). In this study, cgMLST can distinguish the differences and confirm the extremely minute association of these strains of major serotypes. Although they have been grouped into Enteritidis, Typhimurium, and I 4,[5],12:i:- since 2015, we judged the prevalent *Salmonella* was not due to continuous spread of individual strains.

It is worth noting that the traceability is influenced by the use of different cgMLST classification schemes (Li et al., 2008). In several cgMLST scheme protocols, 3002 core loci cgMLST schemes of *Salmonella* promoted in EnteroBase have been widely accepted because of applying the default settings of loci and alleles most likely to be used by many microbiologists to obtain accordant and accurate applications across laboratories and jurisdictions. EnteroBase is well adopted because it covers a large number of isolates’ genomic sequences, and the fixed typing scheme makes the cgMLST results truly comparable among different researchers. EnteroBase covers more than 270,000 *Salmonella* genomic sequences globally and facilitates cgMLST analysis of *Salmonella*. The convenience and pertinence of data collection and collation provide strong data support for pathogen evolution analysis. The fixed typing scheme makes it easy to explore the homologous relationship of individual isolates in the database and to invoke more isolates with certain characteristics. Only by retrieving the isolate information, including time, country or region of isolation, serotype, and MLST and cgMLST typing results of the isolate, could the isolate’s relevant information be obtained. The availability of a web-based analysis platform enables users to conduct cgMLST analysis with minimal local hardware requirements. cgMLST analysis based on thousands of genomic alleles has a higher typing accuracy than MLST. The former was verified to be a more realistic reflection of the evolutionary relationship within the species (Ruppitsch et al., 2015).

High-resolution molecular methods for *Salmonella* typing are not replacing MLST because MLST is relatively convenient for establishing a good relationship with serovars (Kimura, 2018). Comparing evolutionary trees by MLST and cgMLST, the two methods have similar clustering results. However, cgMLST can further distinguish even minor differences between isolates, and the clustering is more detailed. Compared with cgMLST, MLST is generally more discriminatory, but it cannot provide a satisfactory resolution for public health surveillance. Our study also indicates cgMLST is significantly more accurate than MLST, no cgST contains two or more serovars, even I 4,[5],12:i:- and Typhimurium.

According to the evolutionary analysis, the isolates representing the most serovars formed serovar-specific clades in our established neighbor-joining tree except Saintpaul, I 4,[5],12:i:-, and Derby. To explore the genetic and evolutionary relationship among these three serotypes, we further constructed a GrapeTree of the isolates of the three serovars (Figure 4). Only Saintpaul and Derby have multilineages (Yin et al., 2020).

Among the three typing methods, serotyping showed the lowest resolution, and cgMLST had the highest accuracy. Serotyping was based on reactions of antisera to the lipopolysaccharide and flagellar antigens; otherwise, it did not reflect the genetic relatedness between serovars; MLST, based on seven housekeeping genes, was an accurate, reliable typing method, well suited to routine microbial surveillance; WGS-based cgMLST could greatly improve the accuracy of typing and was convenient to share or compare across international labs, so it should be developed in the traceability typing of microorganisms with vitality in the future.

## Conclusion

We investigated 250 *S. enterica* isolates from China during 2004–2019 using serotyping, MLST, and cgMLST. All the *Salmonella* strains were divided into 36 serovars, 43 STs by MLST, and 185 cgSTs by cgMLST. We found the prevalent serovars, STs, and cgSTs of *Salmonella* from different years, regions, and host sources. In addition, we also discovered a novel ST and 182 novel cgSTs. This article clarifies the temporal and spatial distribution characteristics of phenotyping and genotyping diversity of *S. enterica* isolates in China in the recent 16 years. Our results supplement the strain resources, genetic information, and typing data of *Salmonella*; benefit the typing, traceability, determination, and genetic evolution analysis of *Salmonella*; and therefore, promote the level of analysis, monitoring, and prevention and controlling of *Salmonella* in China.

## Data Availability Statement

The datasets presented in this study can be found in online repositories. The names of the repository/repositories and accession number(s) can be found in the article/Supplementary Material.

## Author Contributions

SY, WZ, and LZ were major contributors in writing the manuscript, and conceived and designed the study. SY, LC, and BY contributed materials and resources. CL and XL worked for slide agglutination. All authors read and approved the final manuscript.

## Conflict of Interest

The authors declare that the research was conducted in the absence of any commercial or financial relationships that could be construed as a potential conflict of interest.

## Publisher’s Note

All claims expressed in this article are solely those of the authors and do not necessarily represent those of their affiliated organizations, or those of the publisher, the editors and the reviewers. Any product that may be evaluated in this article, or claim that may be made by its manufacturer, is not guaranteed or endorsed by the publisher.

## Footnotes

1
https://lfz.corefacility.ca/sistr-app/

2
http://enterobase.warwick.ac.uk

## Supplementary Material

Supplementary Table 1 Background information, draft genome, serovars, and genotypes of 250 *Salmonella enterica* isolates. Comment: assembly barcode was obtained by submitting the raw genome data into EnteroBase, and the replacement strain name played an identification role in the established evolutionary tree. SRA numbers were obtained from the NCBI SRA. It is important to note that prefixes such as “QLUL” were added to the strain’s name after submission to EnteroBase in order to obviously distinguish it, so there may be some bias from the SRA database strain names.
